# Critical Systematic Review of 3D Bioprinting in Biomedicine

**DOI:** 10.3390/ijms262411882

**Published:** 2025-12-09

**Authors:** Ilya Klabukov, Victoria Shestakova, Airat Garifullin, Anna Yakimova, Denis Baranovskii, Elena Yatsenko, Michael Ignatyuk, Dmitrii Atiakshin, Peter Shegay, Andrey D. Kaprin

**Affiliations:** 1 National Medical Research Radiological Centre of the Ministry of Health of the Russian Federation, Koroleva st. 4, Obninsk 249036, Russia; 2 Obninsk Institute for Nuclear Power Engineering, National Research Nuclear University MEPhI, Obninsk 249036, Russia; 3 Research and Educational Resource Center for Immunophenotyping, Digital Spatial Profiling and Ultrastructural Analysis Innovative Technologies, Patrice Lumumba Peoples’ Friendship University of Russia (RUDN University), Moscow 117198, Russia; 4 Department of Biomedicine, University of Basel, Petersplatz 1, 4001 Basel, Switzerland; 5 Institute of Systems Biology and Medicine, Russian University of Medicine, Delegatskaya st. 20/1, Moscow 127006, Russia

**Keywords:** artificial organs, bioinks, bioprinting, physiological relevance, regenerative medicine, regenerative surgery, tissue engineering, quantitative morphology

## Abstract

The rapid development of 3D bioprinting technology has not been critically evaluated for its potential clinical applications… The ability of 3D manufacturing to create organ-like structures obscures the fact that the formed grafts are not physiologically relevant. We hypothesize that researchers do not use techniques that allow for the evaluation of the micro-architectonics of formed implants and mainly focus on biocompatibility and commonly observed immunological responses. This study aims to investigate the morphological landscape of the basics of 3D bioprinting through a systematic review of the outcomes of the experimental implantation of bioprinted constructs. A systematic search was conducted in the PubMed database using the following query: (bioprinting OR printing OR bioprinted OR printed OR bioinks) AND (cell OR cells) AND (implantation OR implanted OR in vivo) AND (goat OR porcine OR pig OR swine OR dog OR rabbit OR sheep) NOT (human OR humans). This systematic review evaluated the preformed studies of the in vivo assessment the 3D-bioprinted constructs, and 41 articles meeting the inclusion criteria were selected. We concluded that 3D bioprinting has limited applications for forming living tissue for orthotopic implantation. Additionally, quantitative methods for evaluating the properties and morphological quality of implanted bioprinted constructs have not been developed for tissue engineering applications.

## 1. Introduction

Bioprinting is an additive manufacturing approach to create three-dimensional structures similar to tissues by using biological materials mixed with living cells. Currently, bioprinting tissues and organs is a promising additive technique for creating cell-containing constructs for biomedical applications [1]. Since the invention of the first 3D bioprinter in 1988, which used a jet printer to ink SV-T2 cells [2], 3D bioprinting has remained a promising technique with high expectations. A significant part of the research uses bioprinting to form composite cell-containing tissues or parts of large organs primarily for transplantation purposes [3,4,5] and secondarily for research purposes [6]. The success of extrusion 3D printing in engineering, industry, and “do-it-yourself” home workshops reinforces this [7]. However, the clinical value of extrusion bioprinting in medicine seems overestimated.

Traditionally, bioprinting has been defined as the process of molding a biological object using bioinks—biocompatible materials seeded with cells. With the rise of bioengineering, developing an easy way to form communities of organoids to model interactions in the human body seemed attractive [8,9]. However, these initial hopes have not been realized. This is primarily due to technical difficulties with high-precision cell deposition, controlled cell distribution, vascularization, and innervation in complex, three-dimensional tissues [10]. The primary concern is vessel formation and maturation; consequently, organ vascularization remains the chief obstacle in tissue engineering [11]. Otherwise, insufficient nutrient perfusion, high rates of cell necrosis, and inadequate angiogenesis present additional obstacles [12].

Progress in cell biology has allowed for the development of new biomolecules and cell cultures with application in bioprinting should improve initial outcomes. Indeed, innovations in bioink technology have led to improved printing accuracy [13], preservation of cytocompatibility and mechanical strength [14], and increased cell viability, nutrient diffusion within printed matrices, and integration with body tissues [15,16]. However, this continuous progress masks the lack of significant results regarding the structural and functional integration of implants in vivo.

Cell fate is affected by cell processing, excessive pressure, hypoxic conditions, and the inability to remove metabolites, all of which stimulate the expression of proinflammatory factors. After implantation, the body’s acute or moderate immune response, stimulated by proinflammatory factors, is added. In fact, a critical examination of three-dimensional bioprinting has led to four-dimensional printing [17]. This technology overcomes the limitations of the static and reproducible dynamic properties of living 3D-printed structures [18]. This is because 4D bioprinting uses stimulus-responsive biomaterials and shape-changing effects to create dynamic environments that mimic physiological activity [19]. Additionally, 4D bioprinting utilizes natural cellular forces and stem cell differentiation processes. Stem cells can remodel scaffolds over time, consistent with the concept of 4D bioprinting [18,20,21], which is promising for various biomedical applications, including tissue engineering, drug delivery systems, and in vivo surgery [19,22,23].

One of the famous statements by British physicist Lord Kelvin (1824–1907) is often quoted as follows: “If you cannot measure it, then it is not science”, vividly describes the modern problem of tissue engineering based on 3D and 4D printing approaches [24]. The point is that the benefits of microstructured implantable materials have not been proven because resident cell reactions and connective tissue deposition almost always eliminate the implant, particularly in epithelial tissues [25,26]. We hypothesize that researchers are not using techniques that would allow them to evaluate the micro-architectonics of formed implants. Instead, they primarily study biocompatibility and immunological responses.

This study aims to investigate the morphological landscape of 3D bioprinting basics by systematically reviewing the outcomes of implanting experimental bioprinted constructs.

## 2. Methods

### 2.1. Prisma Guidelines and PROSPERO Registration

This systematic review and meta-analysis were performed and documented following the guidelines outlined in the Preferred Reporting Items for Systematic Reviews and Meta-Analyses (PRISMA) statement [27]. The study was not registered in the International Register of Prospective Systematic Reviews (PROSPERO) due to lack of clinical significance.

### 2.2. Search Request

To identify relevant literature, we conducted a systematic search across the internet database PubMed on 13 October 2025. Our inclusion criteria were based on the PICO framework and focused on English written experimental studies conducted on animals, specifically animal models of goats, pigs, dogs, rabbits, and sheep. In addition, it was critical for this study that the intervention involved the implantation of 3D bioprinted structures containing cells, as well as the availability of histological, morphological, or functional results after implantation. Comparisons between studies and groups were not a requirement for this review. The request in PubMed: “(bioprinting OR printing OR bioprinted OR printed OR bioinks) AND (cell OR cells) AND (implantation OR implanted OR in vivo) AND (goat OR porcine OR pig OR swine OR dog OR rabbit OR sheep) NOT (human OR humans)”. The search was limited to publications from 2010 to October 2025. The titles and abstracts of the references found were independently evaluated by two authors (D.B. and D.A.) working independently. Full texts were obtained for all references that either author considered potentially relevant. In cases of disagreement, a third reviewer was consulted (I.K.) to reach a consensus.

### 2.3. Study Selection

**Figure 1:**
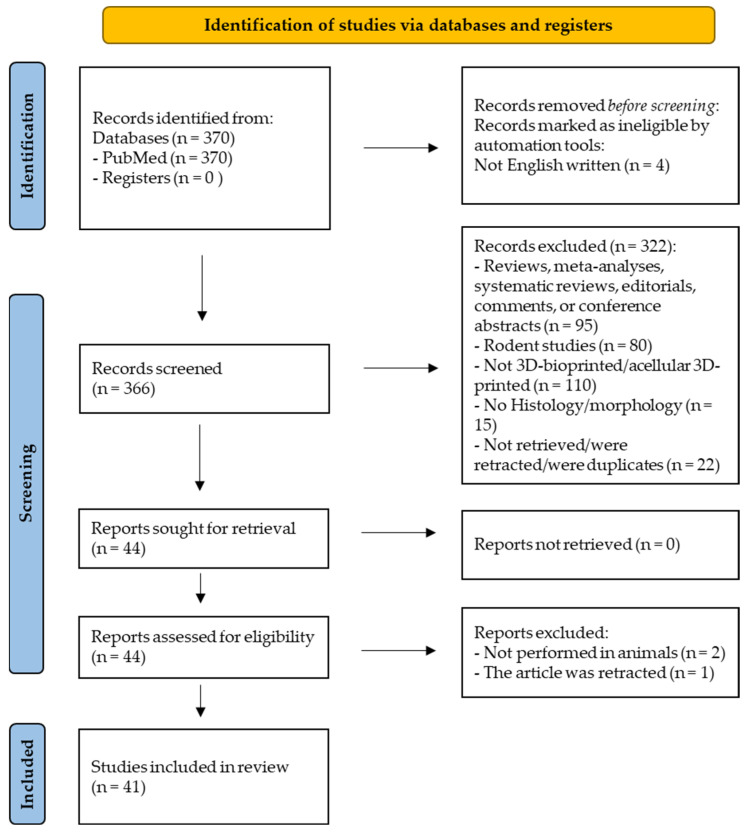
PRISMA search diagram.

We excluded reviews, non-thematic, citations, short communications, case reports, articles written in non-English languages, articles which do not meet the definition of 3D-bioprinting (3DBP), also studies on mice and rats, because the subcutaneous implantation into rats is not relevant and these studies were excluded from the review. The exclusion of articles written in languages other than English was carried out using automated tools—the EasyPubMedicine filter. The PRISMA flowchart diagram (Figure 1) outlines the study selection process.

### 2.4. Outcomes Assessment

We structure the histological and immunohistochemical assessment by structural (collagen deposition, tissue architecture), vascular (VEGF, CD31), immunological (macrophage markers, cytokines assays. Because the bioprinting is based on the micro-structuring of the graft, there is a need for techniques for assessment of microarchitectonics and structural properties of implanted tissues.

### 2.5. Data Extraction

Two researchers from the team (E.Y. and V.S.) independently analyzed the relevant data for each trial. They examined various aspects of the studies, including the authors and publication year, study type, type of bioprinted tissue, host tissue and animal used, technique of in vivo implantation and evaluation of outcomes. Any dis-agreements were resolved through discussion, and if a consensus could not be reached, a final decision was made by a third reviewer (I.K.). The level of agreement between the reviewers was measured using kappa statistics, which showed substantial agreement with a kappa value of 0.88. Data from included studies were extracted into a standardized table. Extracted information included: type of bioprinted tissue, host tissue and animal model, implantation technique, methods for outcome evaluation, and references.

### 2.6. Assessment of Risk of Bias in Individual Studies

Given the review’s objective to evaluate the methodological approaches for assessing bioprinted constructs, a standard risk of bias tool focused on efficacy outcomes (e.g., SYRCLE’s RoB tool) was not deemed fully appropriate. Instead, we developed a focused, criteria-based assessment to evaluate two key domains.

The first area included an assessment of the completeness of results reporting. This focused on the presence or absence of a comprehensive assessment of structural integration and microarchitecture. This was assessed by evaluating the quantitative and qualitative methods used in the studies, as listed in Table 1, specifically those that assess collagen fiber orientation and long-term structural integrity (e.g., polarized light, SHG imaging).

The second area included an assessment of overall methodological rigor. We assessed the presentation of standard methodological elements that impact the reliability of in vivo results, including sample size calculation, randomization of animals to treatment groups, and blinding of outcome assessment during histological analysis. Two reviewers (V.S. and E.Y.) independently applied these criteria to each study.

### 2.7. Synthesis Methods

Due to the heterogeneity in interventions, outcomes, and measurement methods, a meta-analysis was not feasible. The results were synthesized narratively. The findings are presented in structured Table 2 and summarized in the text to describe the characteristics of the included studies, the methodologies used for outcome evaluation, and the key findings related to the review’s objective.

## 3. Results

The systematic search of the PubMed database, covering publications from 2010 to October 2025, initially yielded 370 unique articles. After the initial selection of full texts of primary research articles, 366 papers remained. These articles were sorted according to inclusion/exclusion criteria, and only 41 were selected for full review. The main features and characteristics of each study are presented in Table 1.

Analysis of the included studies revealed several key characteristics that determine the focus of current trends in the experimental and clinical application of bioprinted structures. First, approximately 60% of studies focused on regenerating bone and osteochondral (bone-cartilage) tissues. About 20–25% of experiments aimed to reconstruct cartilage, menisci, and the trachea. The remaining studies focused on the 3D bioprinting of entheses, corneas, dental pulp, gums, and subcutaneous encapsulation systems. Second, the most commonly used animal model was the New Zealand white rabbit, used in over 30 studies. This is likely because their size is optimal for complex surgical procedures such as creating bone defects in the femur or tibia, meniscectomy, and tracheal reconstruction. Additionally, these animals have relatively high regenerative potential, allowing researchers to observe the results of implantation over a controlled period ranging from several weeks to several months. Other animal models included sheep, goats, beagles, and mini-pigs, reflecting a focus on larger animal species that are more clinically relevant and closer to humans.

We also noted that various materials were used to create bioprinted tissues in the studies. Synthetic polymers, such as polycaprolactone (PCL) and polylactic-co-glycolic acid (PLGA), were commonly used to create structural scaffolds. These polymers were combined with natural hydrogels, including gelatin methacrylate (GelMA), fibrin, hyaluronic acid, silk fibroin, alginate, and collagen, to create bioinks that encapsulate cells. Additionally, hydroxyapatite (HA) and β-tricalcium phosphate (β-TCP) ceramics were used in an attempt to increase the osteoconductivity of bone tissue.

Despite their shortcomings, mesenchymal stem cells from various sources remain the most commonly used cell type for seeding the scaffolds [69,70,71]. Other cell types, including chondrocytes, osteoblasts, epithelial cells, and fibroblasts, were also used in some studies. A critical aspect of our study was developing a comprehensive list documenting all methods for evaluating the effectiveness of implanted bioprinted structures. The studies employed various methods, which we categorized and presented in Table 2.

Although the studies listed in Table 1 demonstrate varying degrees of success in tissue regeneration, integration, and functional recovery, our systematic analysis revealed a common flaw in all of them. Specifically, we did not find a single study that included a controlled experiment proving that the microarchitectural structure obtained through 3D bioprinting provides clinically significant functional or structural advantages in vivo compared to non-printed, cell-loaded constructs.

In most studies, researchers attribute positive results, such as the formation of new bone tissue, cartilage matrix, and tracheal epithelium, to the bioactivity of the material, the regenerative potential of the seeded cells, and the healing ability of the host animals. However, despite the complexity of methods for assessing biocompatibility and macroscopic tissue formation, techniques designed to quantitatively assess and confirm the persistence of pre-printed microarchitecture (e.g., collagen fiber alignment and preservation of specific cell patterns) and its physiological advantages after implantation and remodeling were lacking. Therefore, our study’s main finding directly supports developing post-implantation evaluation methods rather than compatible printing methods and bioinks.

## 4. Discussion

The performed systematic review enables us to question whether researchers currently possess conclusive evidence of the functional advantages of bioprinting. This suggests that the field lacks controlled studies directly comparing bioprinted constructs to non-printed, cell-laden equivalents using quantitative metrics of structural and functional outcomes. It also suggests that the authors of these studies are not using histological methods that could reveal structural or functional benefits.

A significant measurement gap plagues the field, as scientists focus on biocompatibility and tissue integration using histological methods while neglecting to study how printed microarchitectonics survive and function inside the body. The host’s wound-healing process disrupts the original arrangement of cells and materials by causing inflammation, attracting resident cells, and producing connective tissue. Ultimately, this leads to resorption and remodeling of the implant. The main advantage of 3D bioprinting is the ability to create a pre-implantation structure [72,73]. However, our review found a critical gap: a widespread lack of techniques to quantitatively confirm the persistence of this pre-printed architecture and demonstrate its direct, causative role in enhancing functional outcomes in vivo compared to non-printed controls. This process also leads to two problems: reduced cell viability due to shear stress during printing [74,75], and the inability to prevent ischemia in the formed core of the structure after implantation due to a lack of immediate vascular integration [12,76]. A justification for the use of bioprinting could be an operating room equipped with “biopens” for the intraoperative repair of tissues, where cells can immediately interconnect with living tissues [77,78].

Additionally, using inappropriate animal models creates confusion and complicates the interpretation of scientific data and evidence of effectiveness. For example, evaluating a trachea-mimicking construct in a subcutaneous model of nude mice does not replicate the complex biomechanical and immunological microenvironment of the orthotopic site, rendering the results physiologically irrelevant [79]. Similarly, using rodent models for skin research creates a false impression [80], primarily due to the organization and distribution of myofibroblasts in rodent skin. Of the skin studies reviewed, four used mice, two used rats (an irrelevant model due to the presence of myofibroblasts in rat skin), and only one used the relevant porcine model. The bioprinting system demonstrated that functional outcomes, such as wound closure and re-epithelialization rates, achieved with in situ bioprinting can be comparable to those obtained using traditional clinical methods such as cell spraying [81]. Despite the fact that this finding is based on the results of just one study and requires further validation, its results highlight the need to critically evaluate whether the complexity of bioprinting translates to a superior clinical benefit in all proposed applications.

Another issue lies in the different approaches to assessing the immune response, which create various difficulties. Interestingly, the immunological responses discussed in the papers addressed only material or seeded cells and were not closely related to the assessment of structural properties. Studies have shown that a multidimensional approach is necessary to evaluate immunological reactions in tissue-engineered constructs [82]. Regardless of the implantation site, bioprinted-patterned grafts seem to not benefit the physiological outcomes after implantation.

Quantitatively assessing the microstructure of tissue-engineered constructs is an important but underdeveloped strategy. Special morphological techniques are required to evaluate structural features in 3D-printed tissues compared to non-printed tissues. Could vascularization and epithelization increase solely due to the initial structure of bioinks? Although spatial pattern assessment can potentially be performed by histological staining, the spatial scale of immunohistochemistry currently lacks the resolution necessary to visualize inner structures. Currently, only a few microscopy methods are able to visualize changes in ECM patterns at a scale of 10–30 µm. Several underutilized techniques with their spatial-resolution potential are presented in Table 3.

The presented data showed that high-resolution polarized light microscopy and second harmonic generation (SHG) imaging are appropriate methods for evaluating tissue morphology. However, these techniques are more complex to perform and require additional equipment in labs. Possible alternatives for high-resolution polarized light and SHG imaging include single-cell sequencing to evaluate alterations in cellular regulation. However, these techniques have only been used preliminarily in cancer research and have not been adopted in regenerative medicine due to the difficulty of studying the heterogeneity of resident cells and the significant differences between novel tissue and normal surrounding tissue.

**Figure 2:**
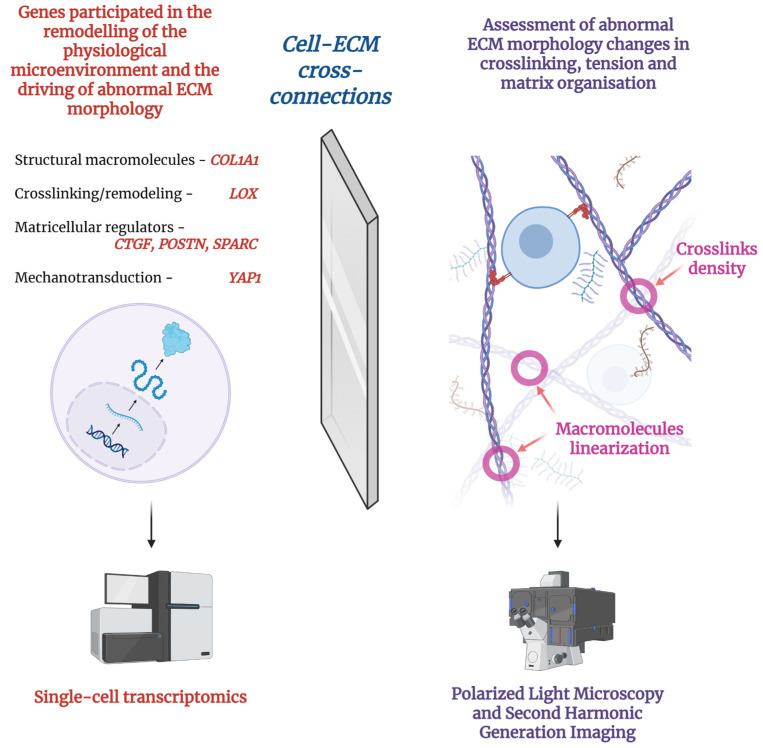
Two-way mirror of cell regulation–ECM interactions shows that changes in cellular regulation and related alterations of ECM properties both could demonstrate the lack of physiological relevance of biocompatible implants. Created with Biorender.com.

The complexity of the morphological evaluation of bioprinted tissues arises from the artificial nature of the constructs and the adaptive responses of resident cells to synthetic or combined materials, which could manifest as altered cell regulation, microarchitectonics, or ECM composition. In this paradigm, ECM composition and orientation could serve as a rough reflection of alterations in cellular regulation in response to environmental changes, demonstrating the physiological relevance of the techniques employed (Figure 2). However, the use of single-cell sequencing is complicated by cell heterogeneity and requires the use of non-standard bioinformatic approaches to identify the transcripts of interest. Therefore, there are no reasonable alternatives to precise morphological evaluations focusing on ECM properties.

Researchers often refer to vascularization as a positive effect of 3D bioconstructions. However, vascularization of bioprinted structures is not fully achieved due to the early migration of monocytes and M1 macrophages, cell death within tissue spheroids, and the lack of physiological relevance of immature constructed tissue. Implanted tissues also do not have time to return to normal metabolic regulation levels. The observed effects of successful vascularization of implants are mainly due to molecular factors of tissue ischemia. Thus, the fundamental issue is that 3D formation is a non-physiological mechanism with no basis in living organisms. In multicellular organisms, the growth and regeneration of tissues is driven by progenitor cells that migrate and proliferate from their specific stem cell niches. Engineering high-potential cell niches is a promising approach for directing specific cellular functions because these niches provide biochemical and mechanical cues that control cell growth, migration, and differentiation [97,98].

Another questionable issue in the assessment of the advantages of 3D bioprinting is the benefit-to-harm ratio. Excessive mechanical stress, ischemia, gas exchange disorders, decreased diffusion of fluids and excretion of metabolites, local overheating, heat shock, and excessive osmotic pressure can devastate cells in bioinks [99,100,101]. Layer-by-layer formation cannot produce oriented cell layers or organoids. Robust fixation of organoids leads to prolonged cell adaptation. The presence of nonlinear effects of radiation modifies materials and alters cellular regulation [102]. Despite their promise, composite materials did not advance compared to existing techniques [103,104]. Table 4 summarizes the most commonly used current bioprinting techniques.

There are a significant number of bioprinting methods and their subtypes with high potential for improving resolution and reducing cell stress. The progress in technological advances in bioprinting does not reflect an understanding of the basic principles of stimulating physiological regeneration or of the engineering principles of synthetic morphogenesis. [116,117]. Unfortunately, the bioprinting approach did not achieve physiological relevance.

The structured bioprinted material is mostly reabsorbed within two to eight weeks and does not have any advantages over the same materials seeded with cells without the mediation of a bioprinter. There is no difference between 3D printing and squeezing the material from a syringe. There are only a few applications for bioprinting that are worth performing, such as intraoperative repair/cell injection or treatment of skin disorders and burns. The benefits of bioprinting are easily outweighed by the capabilities of systems that can maintain long-term cell viability. However, despite the significant shortcomings in the field of 3D bioprinting, there are currently some promising developments that were not the main focus of this review but are aimed at addressing the main critical issues. In particular, this concerns technologies for the development of smart biomaterials for 4D bioprinting, stimulus-responsive materials for creating dynamic biological structures capable of changing shape and function over time [118,119,120], strategies for creating pre-vascularized structures [121,122], and the evolving application of intraoperative bioprinting for direct wound healing [122]. More advanced approaches include the organ bud concept, synthetic morphogenesis, artificial cell chemotaxis, and other in vivo morphogenesis techniques. In conclusion, better regenerative strategies may lie beyond the framework of extruding “bioinks”. Concepts such as organ buds, synthetic morphogenesis, and directed artificial cell chemotaxis use the intrinsic property of cells to self-organize into functional tissue in vivo. Furthermore, while artificial intelligence (AI) research on printing parameter optimization is in its early stages and its impact is minimal [122,123,124], AI-based 3D morphometry could become a valuable asset for properly analyzing key microarchitectural features [125], which are currently lacking in this field.

A limitation of the study was the search principle, including only PubMed data search was included because of the credibility, relevance, and accessibility of the sources indexed. Due to the qualitative nature of the investigation, statistically significant outcomes are not possible. In addition, the review only included large animal studies, so it was not possible to investigate rare precision surgeries performed on rats and mice.

Therefore, the absence of essential in vivo 3D bioprinting studies calls into question the conclusion that precision bioprinting should only be used intraoperatively for target reconstruction or cell injection into damaged tissues, thus eliminating the need for bioprinting machines.

## 5. Conclusions

Recent advancements in micromechanics have enabled the development of 3D bioprinters with great potential. However, the ability of current 3D bioprinting techniques to create living tissues that can easily be implanted is questionable because there are no quantitative methods to evaluate the properties of bioprinted constructs. These properties should include the long-term preservation of the printed microarchitecture, such as collagen fiber alignment, pore geometry, and channel patency; the dynamic evolution of mechanical properties after implantation; and the direct causal relationships between the initial structure and the integration of functional tissue. Based on current evidence from in vivo studies, we conclude that the clinical potential of 3D bioprinting is overestimated. Future studies should focus on developing analytical techniques and designing studies that definitively prove the added value of bioprinting. For example, quantitative morphology techniques could be used to evaluate tissue morphology with high-resolution polarized light and second harmonic generation imaging.

## Figures and Tables

**Table 1:** General methodologies for evaluating outcomes.

General Direction	Methods
Imaging and radiological assessment	1. Microcomputed tomography (MicroCT) was used to assess bone tissue regeneration, providing quantitative data on bone tissue volume, density, and integration with the matrix. 2. Traditional CT and magnetic resonance imaging (MRI) were used for larger structures: the trachea, mandible, and menisci. 3. Scanning electron microscopy (SEM) allowed for ultrastructural analysis of the grafts.
Histological and histomorphometric analysis	1. Hematoxylin and eosin (H&E) staining was the main method used to evaluate tissue morphology. 2. Specialized stains such as safranin-O, Masson’s trichrome, Van Gieson’s stain, and Sirius red were used to detect proteoglycans in cartilage and collagen deposits. 3. Quantitative analysis of histological sections based on histomorphometry allowed measurement of the bone-implant contact ratio, tissue ingrowth area, and glycosaminoglycan (GAG) content.
Immunohistochemistry (IHC)	IHC was aimed at identifying specific tissue components and cell types with the following targets: 1. Collagen types I and II—differentiation of fibrous tissue from hyaline cartilage. 2. CD31—detection of vascular endothelial cells. 3. Osteocalcin—detection of bone tissue. 4. Macrophage markers (CD163, Arg1)—characterization of the immune response.
Biochemical assays	Quantitative determination of DNA, sulfated glycosaminoglycans (s-GAG), and total collagen content was used to assess the biochemical composition and quality of the regenerated tissue.
Biomechanical testing	Mechanical tests, including uniaxial compression, tensile testing, and push-out testing, were performed to investigate and evaluate the functional integration and strength of the tissue.
Immunological and cytokine analysis	Measurement of systemic or local levels of inflammatory cytokines (e.g., IL-1β, IL-6, TNF-α) by ELISA was used to assess the body’s immune response to the implant.

**Table 2:** Cases of orthotopic implantation of 3D-bioprinted grafts.

No.	Type of Bioprinted Tissue	Host Tissue and Animal Used	Technique of In Vivo Implantation	Evaluation of Outcomes	Refs.
1	3D printing polycaprolactone-only scaffold and fibrin/mesenchymal stem cells-coated 3D printing polycaprolactone scaffolds	Femurs and tibias; New Zealand white rabbits	The prepared 3D-printed matrices were implanted into a 10 × 5 mm wedge-shaped defect in the cervical esophagus.	Computed tomography; Histology (hematoxylin and eosin); IHC with desmin staining to assess smooth muscle formation.	[28]
2	Platelet-rich plasma gelatin methacryloyl hydrogel scaffold	Bone and cartilage; Male New Zealand white rabbits	Prepared 3D-printed hydrogel scaffolds were implanted into the defects on the center of the distal articular cartilage of the femur on both sides of the knee.	The macroscopic samples (n = 5) were assessed according to the cartilage repair assessment tool; 3 samples were measured by a Micro-CT scanner; Histology (hematoxylin and eosin staining and Safranin-O fast green staining); IHC (Arg1, CD163, CCR7).	[29]
3	Affinity peptide E7 loaded onto the SF-gelatin scaffolds	Bone and cartilage; Adult male New Zealand white rabbits	3D-printed material was injected into microfractured knee joints	Histology (H&E staining, toluidine blue staining); IHC (collagen type II); Quantification of inflammatory cytokines; Magnetic resonance imaging; Scanning electron microscope.	[30]
4	Porous polyether-ether-ketone cylinder shaped scaffolds with a diameter of 6 mm and a height of 10 mm were either immersed into (hydroxymethyl)aminomethane and dopamine solution or into (hydroxymethyl)aminomethane and MgCl2 solution	Bone and cartilage; Male New Zealand White rabbits	Bilateral femoral condylar bone defects were constructed and scaffold was inserted	Van Gieson’s staining was conducted by Stevenel’s blue and picric acid magenta dye solution. The optical microscope was used to observe sections after staining. The area of bone (stained with dark red) inside the scaffolds was measured and ratio of bone area and total scaffold area was also calculated. The bone-implant contact ratio was measured by calculating the ratio of the length of bone tissue which is directly contacted with the scaffolds to the total length of the surface of scaffolds.	[31]
5	Platelet-derived growth factor-BB, kartogenin, within biomimetic matrix scaffolds (composed of hyaluronic acid methacrylate, lithium phenyl-2,4,6-trimethylbenzoyl phosphinate and meniscal extracellular)	Bone and cartilage; Male New Zealand White rabbits	Major meniscectomy was performed and scaffold was inserted	Macroscopic evaluation using Osteoarthritis Research Society International grading for damaged cartilage and osteophyte development; Biochemical analysis (glycosaminoglycan and total collagen contents of the neomeniscus were assessed using a hydroxyproline assay kit (Nanjing Jiancheng, China) and a tissue GAG Total-Content DMMB Colorimetry Kit (GenMed, Shanghai, China); MRI (regenerations was also analyzed according to the Whole-Organ Magnetic Resonance Imaging Score (WORMS) system); Biomechanical testing (uniaxial mechanical testing machine); Histology (H&E, toluidine blue, sirius red); IHC (collagen 1 and collagen 2).	[32]
6	Calcium phosphate cements loaded with perfluorodecalin and autologous bone marrow	Bone; Adult New Zealand rabbits	A 1 cm long bone defect was created in each ulna after removing the periosteum from the site and 3D-printed specimens were implanted	Histology (H&E); Backscattered scanning electron microscopy; Histomorphometric analysis (assessing bone and soft tissue growth).	[33]
7	Digital light processing3D printed trachea made with silk fibroin and glycidyl methacrylate solution (Sil-MA) loaded with human chondrocytes (labeled with PKH26) and turbinate-derived mesenchymal stem cells (human or rabbit; labeled withPKH67)	Trachea; Male New Zealand white rabbits	A circumferential defect was cut into the anterior tracheal wall using a scalpel. A tissue-engineered trachea was then inserted and anastomosed.	Histology (H&E used to assess the tissue morphology; Safranin O staining used to assess the presence of proteoglycan-rich matrix; Masson’s trichrome staining used to detect collagen production).	[34]
8	Polycaprolactone laryngo-tracheal scaffold loaded with mesenchymal stem cells	Larynx and trachea; Female sheep	Laryngotracheal reconstruction surgery by cervicotomy and laryngofissure with implantation of two scaffolds, one in the laryngeal site and one in the tracheal site	Histology (H&E).	[35]
9	Poly(trimethylene carbonate) with tricalcium phosphate or hydroxyapatite-loaded either with cryogel or bone morphogenetic protein or zoledronic acid (and their different combinations) printed with stereolithography	Bone; Male New Zealand white rabbits (tibial defects) and Belgian white rabbits (cranial defects)	Tibial (near knee joints) and cranial defects (trephine on either side of midline with frontal and parietal bones receiving two defects each)	Micro-CT (quantitative morphometric analysis; mineralized bone volume, tissue mineralization, and scaffold-bone integration were assessed); Histology (H&E, Masson’s trichrome staining).	[36]
10	3D printed polylactic acid system termed neovascularized implantable cell homing and encapsulation (filled with 300 μL platelet-rich plasma alginate hydrogel)	Subcutaneous tissue; Yucatan minipigs	Implants were implanted in the dorsum of Yucatan minipigs	Monotonic axial central compression tests; Histology (H&E and Picrosirius staining).	[37]
11	Gelatin methacrylate and long-chain poly(ethylene glycol) diacrylate blended and laden with L929 mouse fibroblast cells and rabbit corneal epithelial cells	Eye cornea; Adult male rabbits	Anterior lamellar keratoplasty was performed. Then scaffolds were punched with a trephine and carefully put on the corneal defect area with the printed epithelia layer facing upwards.	Slit lamp monitoring; Histology (H&E); IHC (cytokeratin 3, collagen type I, lumican, and alpha smooth muscle actin); Gene expression comparison (KERA, ALDH, AQP1 genes).	[38]
12	Hydroxyapatite/poly (lactic-co-glycolic acid) (HA/PLGA) three-dimensional porous scaffolds	Femoral condyle; New Zealand White rabbits	Grafts were implanted in the distal condyle of the femur on the right and left sides, in which a cylindrical bone defect (diameter—5.5 mm, depth—6 mm) was drilled.	Three-dimensional analysis using the vivaCT40 micro-CT system; Microtomographic data sets using direct 3D morphometry (visual assessment of structural images of bones and skeletons and morphometric parameters); Histology.	[39]
13	A rubber-like thermoplastic co-polyester, Flexifill, used to print template loaded with adipose-derived mesenchymal stem cells	Cartilage; Male New Zealand white rabbits	The incision was made along the auricular central arteriovenous branch and filled with a 3D template.	Micro-CT; Atomic force microscopy	[40]
14	Polycaprolactone scaffolds laden with rabbit auricular chondrocytes	Cartilage; Male New Zealand white rabbits.	A perichondrium pocket was designed (circle shape biopsy of elastic cartilage of 1 cm of diameter was extracted without penetrating the lateral skin).	Histology (glycosaminoglycan content was visualized by Alcian Blue-Safranin O staining); IHC (type I and type II collagens); Gene expression (ACAN, COL1A1, COL2A1, COL10A1, and SOX9); Scanning electron microscopy; Uniaxial compression tests	[41]
15	Polylactic acid glycolic acid scaffold with bone morphogenetic protein-9 and P-15 peptide hydrogel	Bone; Japanese big ear rabbits	The hole was created in the bone above the articular surface of the lateral condyle of the femur, then filled with the scaffold.	MicroCT; Gene expression (ALP, COL-1, OCN, RUNX2, and Sp7)	[42]
16	Osteogenic part (3D printed polycaprolactone crosslinked with dopamine; Chondrogenic part (silk fibroin loaded with bone morphogenetic protein 2)	Bone and cartilage; Mature New Zealand white rabbits	Defects were made in the acetabulum then filled with the implants	Histology (H&E), Safranin O-Fast Green, toluidine blue, and Masson’s trichrome); IHC (Collagen II, assessed semi-quantitatively with the use of a microscope and Image-Pro Plus software (Media Cybernetics, https://mediacy.com/image-pro/ accessed on 5 December 2025); the relative density (integral optical density/area) was calculated to semi-quantify the content of collagen II); CT; MRI; Unconfined compressive strength.	[43]
17	Bone marrow-derived mesenchymal stromal cells and adipose tissue-derived stem cells loaded onto 3D- printed scaffolds	Bone; Mature New Zealand male rabbits	Mandibular bone defect was created, then filled with the scaffold	Micro-CT.	[44]
18	Poly(caprolactone) scaffold loaded with goat autologous auricular cartilage cells	Trachea; Shanghai white goats	Goat native trachea (covering four full cartilage rings) was surgically excised and replaced by tissue-engineered trachea	Bronchoscopy; CT; Histology (H&E, toluidine blue, and Safranin O); IHC (type II collagen, cytokeratin); TUNEL staining (In Situ Cell Death Detection Kit; score for apoptotic cells).	[45]
19	Nanoporous hydroxyapatite implant loaded with autologous bone marrow stromal cells	Bone; Mature Beagles	Bone defect was created in the mandibules; the cell scaffold composite was placed in the defects and firmly fixed with titanium plates and titanium nails.	CT and micro-CT; Electron microscopy; Histology (methylene blue-acid fuchsin).	[46]
20	β-tricalcium phosphate /poly (lactic-co-glycolic acid) scaffold was loaded with HA15 (osteogenesis-promoting drug)	Bone; New Zealand white rabbits	Radial defects were made, then filled with the scaffold.	Micro-CT (assessing bone volume/total volume, bone mineral density, trabecular thickness, and the structural model index); Microfil-angiography.	[47]
21	3D printed porous metal scaffolds filled with hyaluronic acid-hydrazide and hyaluronic acid-aldehyde, Infliximab and seeded with adipose-derived mesenchymal stem cells	Bone; 30-week-old female New Zealand white rabbits	A cylindrical defect in the femoral condyle was produced using a drill, the scaffold was implanted into the defect	Histology (H&E and Masson staining; qualitative analysis of bone in-growth); In vivo measurement of cytokines in serum at 3 months (IL-1β, IL-6, TNF-α, and OVA-Ab); Micro-CT; Push out test.	[48]
22	Hydroxyapatite/β tricalcium phosphate/silk fibroin scaffolds seeded with MC3T3-E1 cells (a mouse monoclonal preosteoblastic cell line from C57BL/6 mouse calvaria).	Bone; 6-month old New Zealand white rabbits	Scaffolds implanted into the proximal tibia	Micro-CT; Van Gieson’s staining without decalcification; IHC (Fluorescent double-labeling detection of subcutaneous injected tetracycline and calcein); Axial loading test in alive animals.	[49]
23	Human dental pulp stem cells laden onto the gelatin methacrylate scaffold.	Teeth; Female Panama minipigs	Scaffolds were implanted into the dental root canals, the corona was filled with resin.	Histology (H&E)	[50]
24	Poly (ε-caprolactone) scaffolds with fish collagen and the osteogenic abalone intestine gastro-intestinal digests from Haliotis discus hannai seeded with mouse mesenchymal stem cells.	Bone; Rabbits (not elaborated)	A bone tunnel of 2.1 mm in diameter created in the center of each tibia bone where the scaffolds were later implanted.	X-ray; Micro-CT (rate of new bone volume); Histology (H&E).	[51]
25	Poly(ε-caprolactone) molten to fabricate scaffolding structure with synovium-derived mesenchymal stem cells-laden hydrogel encapsulating poly(lactic-co-glycolic acid) microspheres carrying transforming growth factor, beta 3 or connective tissue growth factor and magnesium ions	Bone and cartilage; Rabbits (not elaborated).	Total medial meniscus was dissected, and the engineered meniscus construct was transplanted in situ.	IHC (CD31, alpha smooth muscle actin, collagen I, collagen II).	[52]
26	Bi-layer scaffold: an interleukin-4-loaded radially oriented gelatin methacrylate scaffold printed with digital light processing in the upper layer and a porous polycaprolactone and hydroxyapatite scaffold printed with fused deposition modeling in the lower layer	Bone; Adult male New Zealand white rabbits.	Osteochondral cylindrical cartilage defects were created on the patellar groove; the bi-layer scaffolds were then implanted.	Histology (safranin-O); Immunohistochemistry (COL2); Tensile-compressive testing; Micro-CT.	[53]
27	Poly(ε-caprolactone) scaffold as a backbone, followed by injection with the meniscus extracellular matrix	Bone and cartilage; Skeletally mature New Zealand White rabbits	Total medial meniscectomy except 5% of the external rim where scaffold was implanted	Meniscus covering rate; Histology (H&E; toluidine blue); IHC (collagen I, collagen II); GAG content assay; Compressive and tensile strengths; X-ray; MRI.	[54]
28	Bone marrow-derived mesenchymal stem cells transfected with a recombinant adenovirus encoding bone morphogenic protein 12 and loaded onto polylactic-co-glycolic acid scaffolds	Tendon and bone; Adult rabbits (not elaborated)	Scaffolds placed in the interface between the supraspinatus tendon and bone	Biomechanical tension testing; Histology (H&E); Modified tendon maturing score (histological signs of fibrous tissue ingrowth, fibrocartilage cells and a tidemark).	[55]
29	Human tonsil-derived mesenchymal stem cells-laden polycaprolactone and beta-tricalcium phosphate scaffold	Bone 12-week old male New Zealand white rabbits	Bone defect in the middle portion of the inferior mandibular margin made with a surgical drill and osteotome and filled with scaffold	Conventional CT; Micro-CT (region of interest between the proximal and distal fixing screws within the scaffold); Mechanical compression assessment; Histology (H&E; Masson’s trichrome staining); IHC (CD31; anti-nuclei antibody).	[56]
30	Polycaprolactone beta-tricalcium phosphate scaffolds placed in porcine neutralized bone decellularized extracellular matrix	Bone; 12-week old male New Zealand white rabbits		Micro-CT (radiodensity analysis used to measure bone volume and density); Histology (H&E; Masson’s trichrome staining; Von Kossa staining).	[57]
31	Hydroxyapatite loaded with bone morphogenic protein-2 and vascular endothelial growth factor.	Bone; New Zealand white rabbits	The scaffolds were implanted into the calvarian defects.	Micro-CT (bone volume to tissue volume, trabecular number); Histology (H&E); IHC (lectin; collagen type I)	[58]
32	Poly (vinyl alcohol) loaded with rabbit meniscal decellularized extracellular matrix.	Bone; Female New Zealand white rabbits	Circular defect was created in the central part of lateral meniscus and replaced with scaffold.	Macroscopic evaluation; Micro-MRI; Histology (H&E, toluidine blue, Safranin O); IHC (Collagen II).	[59]
33	Rabbit bone marrow-derived mesenchymal stem cells (bMSC) and rabbit respiratory epithelial cells-laden polycaprolactone formed artificial trachea	Trachea; Male, 3-month-old New Zealand white rabbits	Half-pipe-shaped partial tracheal resection that was later replaced with an artificial trachea	Bronchoscopy; Conventional CT; Histology (H&E); Safranin-O/fast green).	[60]
34	Bone morphogenetic protein-2-loaded onto bioactive glass S53P4/ polycaprolactone scaffold.	Bone and cartilage; 5-month-old New Zealand rabbits	Outer 1/3 of the external auditory canal and the bone of the lateral wall of the auditory bulla area were abraded. Scaffold was implanted	Otolaryngoscopy; Micro-CT (region of interest, and the total volume, new bone volume, new bone volume fraction.	[61]
35	Poly(ε-caprolactone) scaffold laden with rabbit bone-marrow derived mesenchymal stem cells.	Bone and cartilage; Skeletally mature, male New Zealand White rabbits	Total meniscectomy was performed by resecting the medial meniscus sharply along the periphery and detaching it from its anterior and posterior junction. Scaffold was implanted	Gross evaluation (implant integration, implant position, horn position, shape, presence of tears in the implant, implant surface, implant size, tissue quality, and condition of the synovia); Synovial fluid analysis (IL-1, TNF-a; ELISA); Histology (H&E and toluidine blue); IHC (picrosirius red, collagen I, collagen II); Biomechanical testing (tensile testing, elastic modulus, confined compression testing).	[62]
36	Rabbit-ear primary chondrocytes-alginate bio-ink printed with polycaprolactone.	Cartilage; 12-week-old male New Zealand white rabbits	Circular defects were created using an 8 mm biopsy puncher in the proximal region of the rabbit ears	Histology (H&E; Alcian Blue); IHC (green fluorescent dye, labeled chondrocytes were observed under the confocal laser scanning microscope).	[63]
37	Beagle autologous bone and a bone marrow-derived mesenchymal stem cell hydrogel.	Bone; Beagle dogs	2 cm diameter full-thickness cranial defect was made in the flat part of the head which was 2.5 cm to the right of the midline and 2.5 cm behind the anterior fontanelle. Scaffolds were implanted 3 months after the operation	Micro-CT (bone volume per tissue volume, trabecular number, trabecular spacing); CT; Histology (H&E, Masson’s trichrome staining, Safranine O-Fast green); IHC (TNF-α, IL-1β, collagen I, collagen II, aggrecan, osteocalcin, CD31, Col1, Col2, CD105, CD90, stromal cell-derived factor 1); SDF1 (ELISA).	[64]
38	Acellular porcine dermal matrix gelatin, sodium alginate combined with beagle dog gingival fibroblasts.	Bone and gingiva; Healthy male Beagles	Complexes were transplanted into the mandibular gingival defects	Histology (H&E, Masson and Sirius Red staining); IHC(collagen I, collagen III, vascular endothelial- derived growth factor-A).	[65]
39	Multi-layer structure including PCL layers, hydrogel layer with rabbit nasal epithelial cells and hydrogel layer with rabbit auricular cartilage cells	Trachea; Mature male New Zealand White rabbits	The ventral portion of the trachea was cut into a semi-cylindrical shape measuring approximately 1.5 × 1.5 cm, and the artificial trachea was put into place	X-ray; Respiration pattern assessment; Histology (H&E, Masson’s trichrome, and safranin O).	[66]
40	Poly-L-lactic acid/hydroxyapatite scaffold printed with rabbit bone marrow mesenchymal stem cells -laden and rabbit periosteum-derived stem cells-laden gelatin methacryl hydrogel	Bone; Male New Zealand White rabbits	Symmetrical 8 mm-diameter hole-shaped bone defects were established along the midline of the sutura cranii with a circular drill and filled with scaffolds	Micro-CT (regenerated bone volume, bone volume/total volume, trabecular number, trabecular thickness, and trabecular spacing); Histology (H&E, Masson’s trichrome); IHC (osteocalcin)	[67]
41	Rabbit bone marrow stem cells were used in decellularized bone extracellular matrix (loaded with bone morphogenetic protein-2) and in decellularized cartilage extracellular matrix (loaded with transforming growth factor-beta) combined with silk fibroin bioinks, polycaprolactone was used as a scaffold.	Bone; New Zealand white rabbits weighted 2.0–3.0 kg	Osteochondral defects were caused on the patellar groove of a right knee joint, scaffolds were implanted into the defects.	Histology (safranin O and Masson’s trichrome); IHC (collagen type II, osteocalcin); Biochemical analysis (collagen and sulfated- glycosaminoglycan).	[68]

**Table 3:** Methods for assessment of directional and structural patterns based on collagen fiber orientation.

Histological Technique	Spatial Scale Resolution, μm	Angular Resolution	Refs.
Van Gieson	1–10	Low (Qualitative)	[83,84,85]
Masson	1–10	Low (Qualitative)	[86,87]
Silver staining	1–10	Low (Qualitative)	[88,89]
Polarized light	1–100	High	[90,91,92]
Terahertz spectroscopy	10–100	Moderate	[93]
Second Harmonic Generation (SHG) Imaging	<1 (sub-micron)	Very High	[94,95,96]

**Table 4:** Features and limitations of bioprinting techniques.

Technique	Principle	Features of the Morphology of Bioprinted Structures	Effects on the Cell Fate and Physiological Relevancy	Refs.
Inkjet Bioprinting	A printing head is used to eject droplets of bioink onto a substrate.	Initial additive placement of various types of spheroids or tissue compartments with high spatial resolution.	Cell viability can be affected by shear stress and heat.	[105,106,107,108]
Laser-Assisted Bioprinting	A high-pressure bubble is generated using laser pulses, which propels small droplets of bioink toward a receiving substrate.	High printing precision and spatial resolution of formed constructs.	Cell regulation could be affected by combined irradiation and impulse pressure.	[106,109,110]
Stereolithography or Digital Light Processing Bioprinting	Photosensitive bioinks are photopolymerized by light (usually UV or visible) in a layer-by-layer process.	Extremely high resolution and smooth surface finish. Rapid printing time. Restricted to photo-crosslinkable bioinks.	Potential phototoxicity to cells if not carefully controlled.	[109,111,112]
Extrusion Bioprinting	Pneumatic, piston, and screw-driven systems are used to extrude continuous strands of bioink through a nozzle.	The resolution is lower compared to methods using inkjet or laser-assisted methods.	Shear stress occurs on cells during extrusion.	[105,113]
Electrospinning or electrohydrodynamic bioprinting	Fine fibers or droplets are drawn from a polymer solution or bioink using electric fields.	Nanofibrous scaffolds that mimic the ECM can be created.	Cell viability is often limited due to electric fields and solvent use.	[114,115]

## Data Availability

The original contributions presented in this study are included in the article. Further inquiries can be directed to the corresponding author(s).

## Abbreviations

The following abbreviations are used in this manuscript:

AI	Artificial Intelligence
IHC	Immunohistochemistry
H&E	Hematoxylin and Eosin
SHG	Second Harmonic Generation
